# Prevalence of chronic respiratory disease in urban and rural Uganda

**DOI:** 10.2471/BLT.18.216523

**Published:** 2019-03-26

**Authors:** Trishul Siddharthan, Matthew Grigsby, Brooks Morgan, Robert Kalyesubula, Robert A Wise, Bruce Kirenga, William Checkley

**Affiliations:** a Division of Pulmonary and Critical Care, School of Medicine, Johns Hopkins University, 1830 E. Monument Street, Room 555, Baltimore, Maryland, MD 21287, United States of America.; b College of Health Sciences, Makerere University, Kampala, Uganda.

## Abstract

**Objective:**

To determine the prevalence of chronic respiratory diseases in urban and rural Uganda and to identify risk factors for these diseases.

**Methods:**

The population-based, cross-sectional study included adults aged 35 years or older. All participants were evaluated by spirometry according to standard guidelines and completed questionnaires on respiratory symptoms, functional status and demographic characteristics. The presence of four chronic respiratory conditions was monitored: chronic obstructive pulmonary disease (COPD), asthma, chronic bronchitis and a restrictive spirometry pattern.

**Findings:**

In total, 1502 participants (average age: 46.9 years) had acceptable, reproducible spirometry results: 837 (56%) in rural Nakaseke and 665 (44%) in urban Kampala. Overall, 46.5% (698/1502) were male. The age-adjusted prevalence of any chronic respiratory condition was 20.2%. The age-adjusted prevalence of COPD was significantly greater in rural than urban participants (6.1 versus 1.5%, respectively; *P* < 0.001), whereas asthma was significantly more prevalent in urban participants: 9.7% versus 4.4% in rural participants (*P* < 0.001). The age-adjusted prevalence of chronic bronchitis was similar in rural and urban participants (3.5 versus 2.2%, respectively; *P* = 0.62), as was that of a restrictive spirometry pattern (10.9 versus 9.4%; *P* = 0.82). For COPD, the population attributable risk was 51.5% for rural residence, 19.5% for tobacco smoking, 16.0% for a body mass index < 18.5 kg/m^2^ and 13.0% for a history of treatment for pulmonary tuberculosis.

**Conclusion:**

The prevalence of chronic respiratory disease was high in both rural and urban Uganda. Place of residence was the most important risk factor for COPD and asthma.

## Résumé

**Objectif:**

Déterminer la prévalence de maladies respiratoires chroniques en zones urbaines et rurales en Ouganda et identifier les facteurs de risque associés à ces maladies.

**Méthodes:**

L'étude de prévalence basée sur une population incluait des adultes âgés de 35 ans ou plus. Tous les participants ont été soumis à un test de spirométrie conformément aux directives standards et ont rempli un questionnaire sur leurs symptômes respiratoires, leur état fonctionnel et leurs caractéristiques démographiques. La présence de quatre maladies respiratoires chroniques a été contrôlée: bronchopneumopathie chronique obstructive, asthme, bronchite chronique et modèle de spirométrie restrictive.

**Résultats:**

Au total, 1502 individus (âge moyen: 46,9 ans) ont obtenu des résultats de spirométrie acceptables, reproductibles: 837 (56%) à Nakaseke, en zone rurale, et 665 (44%) à Kampala, en zone urbaine. Dans l'ensemble, 46,5% (698/1502) étaient des hommes. La prévalence ajustée selon l'âge de toutes les maladies respiratoires chroniques était de 20,2%. La prévalence ajustée selon l'âge de la bronchopneumopathie chronique obstructive était considérablement plus élevée chez les participants ruraux que chez les participants urbains (6,1 contre 1,5%, respectivement; *P* < 0,001), tandis que la prévalence de l'asthme était considérablement plus élevée chez les participants urbains: 9,7% contre 4,4% pour les participants ruraux (*P* < 0,001). La prévalence ajustée selon l'âge de la bronchite chronique était similaire chez les participants ruraux et urbains (3,5 contre 2,2%, respectivement; *P* = 0,62), de même que la prévalence d'un modèle de spirométrie restrictive (10,9 contre 9,4%; *P* = 0,82). Dans le cas de la bronchopneumopathie chronique obstructive, la fraction étiologique du risque était de 51,5% pour le lieu de résidence rural, 19,5% pour l'usage du tabac, 16,0% pour un indice de masse corporel < 18,5 kg/m^2^ et 13,0% pour un antécédent de traitement de la tuberculose pulmonaire.

**Conclusion:**

La prévalence de maladies respiratoires chroniques était élevée à la fois en zone rurale et en zone urbaine en Ouganda. Le lieu de résidence était le facteur de risque le plus important pour la bronchopneumopathie chronique obstructive et l'asthme.****

## Resumen

**Objetivo:**

Determinar la prevalencia de las enfermedades respiratorias crónicas en las zonas urbanas y rurales de Uganda e identificar los factores de riesgo de estas.

**Métodos:**

El estudio transversal basado en la población incluyó adultos de 35 años o más. Todos los participantes se evaluaron por espirometría de acuerdo con las directrices estándar y completaron cuestionarios sobre los síntomas respiratorios, el estado funcional y las características demográficas. Se monitorizó la presencia de cuatro afecciones respiratorias crónicas: enfermedad pulmonar obstructiva crónica (EPOC), asma, bronquitis crónica y un patrón de espirometría restrictivo.

**Resultados:**

En total, 1502 participantes (edad media: 46,9 años) tuvieron unos resultados de espirometría aceptables y reproducibles: 837 (56 %) en la zona rural de Nakaseke y 665 (44 %) en la zona urbana de Kampala. En total, el 46,5 % (698/1502) eran hombres. La prevalencia ajustada por edad de cualquier afección respiratoria crónica fue de 20,2 %. La prevalencia de la EPOC ajustada por edad fue significativamente mayor en los participantes rurales que en los urbanos (6,1 frente a 1,5 %, respectivamente; p < 0,001), mientras que el asma fue significativamente más frecuente en los participantes urbanos: 9,7 % frente a 4,4 % en los participantes rurales (p < 0,001). La prevalencia ajustada por edad de la bronquitis crónica fue similar en los participantes rurales y urbanos (3,5 % frente a 2,2 %, respectivamente; p = 0,62), al igual que la de un patrón espirométrico restrictivo (10,9 % frente a 9,4 %; p = 0,82). Para la EPOC, el riesgo atribuible a la población fue del 51,5 % para la residencia rural, del 19,5 % para el tabaco, del 16,0 % para un índice de masa corporal inferior a 18,5 kg/m^2^ y del 13,0 % para un historial de tratamiento de la tuberculosis pulmonar.

**Conclusión:**

La prevalencia de las enfermedades respiratorias crónicas era alta tanto en las zonas rurales como en las urbanas de Uganda. El lugar de residencia fue el factor de riesgo más importante para la EPOC y el asma.

## ملخص

**الغرض:**

تحديد مدى انتشار أمراض الجهاز التنفسي المزمنة في المناطق الحضرية والريفية في أوغندا، وتحديد عوامل الخطر لهذه الأمراض.

**الطريقة:**

دراسة لقطاعات سكانية متعددة شملت الكبار ممن يبلغون 35 عاماً أو أكبر. تم تقييم جميع المشاركين عن طريق قياس التنفس وفقا للمبادئ التوجيهية القياسية والاستبيانات المكتملة على أعراض الجهاز التنفسي، والحالة الوظيفية، والخصائص الديموغرافية. تم رصد وجود أربع حالات تنفسية مزمنة: مرض الانسداد الرئوي المزمن (COPD)، والربو، والتهاب الشعب الهوائية المزمن، ونمط قياس التنفس المقيد.

**النتائج:**

إجمالاً، كان لدى 1502 مشاركًا (متوسط العمر: 46.9 عامًا) نتائج قياس تنفس مقبولة ومتكررة: 837 (56٪) في منطقة ناكاسيكي الريفية و665 (44٪) في كامبالا الحضرية. وعموما، كان 46.5٪ (698/1502) من الذكور. كان معدل انتشار أي حالة تنفسية مزمنة ذات صلة بالعمر هو 20.2٪. كان معدل انتشار مرض الانسداد الرئوي المزمن ذي الصلة بالعمر أكبر بكثير في المشاركين من المناطق الريفية عنه في المناطق الحضرية (6.1٪ مقابل 1.5٪، على التوالي؛ نسبة الاحتمال < 0.001)، في حين أن الربو كان أكثر شيوعاً بشكل كبير في المشاركين من المناطق الحضرية: 9.7٪ مقابل 4.4٪ في المشاركين من المناطق الريفية (نسبة الاحتمال < 0.001). كان معدل انتشار التهاب الشعب الهوائية المزمن ذي الصلة بالعمر مشابهاً في المشاركين من المناطق الريفية والمناطق الحضرية (3.5٪ مقابل 2.2٪، على التوالي؛ نسبة الاحتمال = 0.62)، وكذلك كان الحال في نمط قياس التنفس المقيد (10.9٪ مقابل 9.4٪؛ نسبة الاحتمال = 0.82). بالنسبة لمرض الانسداد الرئوي المزمن، كان الخطر المنسوب للسكان هو 51.5٪ للإقامة في مناطق ريفية، و19.5٪ لتدخين التبغ، و16.0٪ لمؤشر كتلة الجسم الأكبر من 18.5 كجم/م2، و13.0٪ لوجود تاريخ سابق لعلاج السل الرئوي.

**الاستنتاج:**

كان معدل انتشار الأمراض التنفسية المزمنة مرتفعاً في كل المناطق الريفية والحضرية بأوغندا. كان مكان الإقامة أهم عامل خطر لمرض الانسداد الرئوي المزمن والربو.

## 摘要

**目的:**

旨在确定乌干达城乡地区的慢性呼吸道疾病患病率，并确定这些疾病的危险因素。

**方法:**

这项基于人口的横断面研究包括年龄在 35 岁或以上的成年人。所有男性均按照标准指南进行肺活量测定，并完成了调查问卷上有关呼吸症状、功能状态和人口学特征的相关问题。监测四种慢性呼吸系统疾病：慢性阻塞性肺病 (COPD)、哮喘、慢性支气管炎和限制性通气功能障碍的患病情况。

**结果:**

共有 1502 名参与者（平均年龄：46.9 岁）有可接受和可重复的肺活量测定结果：837 名 (56%) 来自 Nakaseke 农村地区，665 名 (44%) 来自坎帕拉城区。总体上，46.5% (698/1502) 为男性。所有的慢性呼吸系统疾病的年龄标化患病率为 20.2%。农村参与者的慢性阻塞性肺病年龄标化患病率明显高于城市参与者（分别为 6.1% 和 1.5%；*P* < 0.001），然而哮喘的年龄标化患病率在城市更高：分别为城市参与者 9.7% 和农村参与者 4.4% （*P* < 0.001）。农村和城市参与者的慢性支气管炎年龄标化患病率相近（分别为 3.5% 和 2.2%；*P* = 0.62），限制性通气功能障碍年龄标化患病率亦是相近（分别为 10.9% 和 9.4%；*P* = 0.82）。对于慢性阻塞性肺病而言，城市居民的人群归因危险度为 51.5%，吸烟者为 19.5%，身体质量指数 < 18.5 kg/m2 的人群归因危险度为 16.0%，有肺结核治疗史的人群归因危险度为 13.0%。

**结论:**

乌干达城乡地区的慢性呼吸道疾病患病率均很高。居住地是慢性阻塞性肺病和哮喘的首要危险因素。

## Резюме

**Цель:**

Определить распространенность хронических респираторных заболеваний в сельских и городских районах Уганды и выявить факторы риска для этих заболеваний.

**Методы:**

Проводилось популяционное перекрестное исследование среди взрослого населения в возрасте от 35 лет. Все участники оценивались посредством спирометрии в соответствии со стандартными рекомендациями. Они заполняли анкеты о респираторных симптомах, своем функциональном состоянии и указывали демографические характеристики. Отслеживалось наличие четырех хронических респираторных заболеваний: хронической обструктивной болезни легких (ХОБЛ), астмы, хронического бронхита и рестриктивных нарушений.

**Результаты:**

Всего было обследовано 1502 участника (средний возраст составил 46,9 года), которые продемонстрировали приемлемые и воспроизводимые результаты спирометрии: 837 (56%) в сельском районе Накасеке и 665 (44%) в городском районе Кампала. Из них 46,5% (698 из 1502 человек) составляли мужчины. Распространенность любого из исследуемых хронических респираторных заболеваний с учетом поправки на возраст составила 20,2%. Распространенность ХОБЛ с учетом поправки на возраст была значительно выше в сельской местности, чем среди участников-горожан (6,1 и 1,5% соответственно, *P* < 0,001), тогда как астма намного заметнее преобладала среди горожан: 9,7 против 4,4% в сельской местности (*P* < 0,001). Распространенность хронического бронхита с поправкой на возраст почти не отличалась в сельской и городской местности (3,5 и 2,2% соответственно, *P* = 0,62), то же самое наблюдалось и для рестриктивных нарушений (10,9 и 9,4%, *P* = 0,82). Популяционный риск, приписываемый фактору ХОБЛ, составлял 51,5% для проживающих в сельской местности, 19,5% для курящих табак, 16,0% для участников с ИМТ менее 18,5 кг/м^2^ и 13,0% для участников с перенесенным туберкулезом легких в анамнезе.

**Вывод:**

Распространенность хронических респираторных заболеваний и в сельской и в городской местности Уганды оказалась высокой. Наиболее существенным фактором риска для ХОБЛ и астмы оказалось место проживания.

## Introduction

Chronic respiratory disease affects one billion people worldwide and is a leading cause of death.^1^ Most forms of the disease are noncommunicable, such as chronic obstructive pulmonary disease (COPD), asthma, chronic bronchitis, occupational lung disease and pulmonary hypertension. Currently, the associated morbidity and mortality principally occur in low- and middle-income countries, where the disease burden is expected to rise as rapid economic gains lead to increases in longevity, industrialization and tobacco consumption.^1^^–^^3^ In addition to its impact on individuals, this epidemiological transition has substantial direct and indirect economic implications.^4^

Urbanization has been associated with noncommunicable diseases, including chronic respiratory disease,^5^^,^^6^ and is expected to increase as virtually all future population growth will be concentrated in urban areas.^7^ There are particular concerns about sub-Saharan Africa, where rapid urbanization and population growth are coupled with an inadequate health infrastructure and poor urban planning.^7^ Little is known about the association between urbanization and the shifting burden of chronic respiratory disease in low- and middle-income countries, particularly those in sub-Saharan Africa. Previous studies have examined the prevalence of COPD in either rural or urban settings,^8^^–^^10^ but none has investigated how the pattern of chronic respiratory disease varies across different residential settings, with the aim of evaluating the impact of the urban environment. Moreover, understanding of the population attributable risk (PAR) for different chronic respiratory diseases in African populations is limited. Nevertheless, COPD has been closely linked to household air pollution in rural areas in low- and middle-income countries and the disease burden is expected to increase in urban areas with the growing prevalence of tobacco smoking.^2^

The primary aim of this study was to examine variations in the prevalence of different chronic respiratory diseases and their attributable risk factors between urban and rural Uganda. In both settings, we assessed the respiratory symptoms and functional status of individuals with obstructive lung disease.

## Methods

**Fig. 1:**
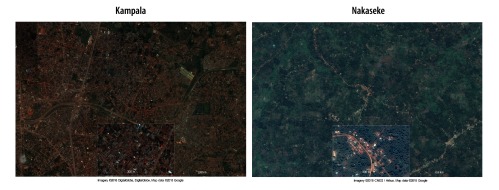
Kampala (urban) and Nakaseke (rural) enumeration areas, study of chronic respiratory disease, Uganda, 2015–2016

Note: In each image, the highlighted area represents one of the 25 enumeration areas selected randomly in each setting with the probability of selection proportional to the population size of the enumeration area. Maps obtained from Google Earth version 9.0 (2017 Apr 8).^13^ Kampala, Uganda: 0°20’51”N, 32° 35’15”E, Eye alt 1,176m. CNES/Airbus 2018. Nakaseke, Uganda: 0°41’59”N, 32° 24’42”E, Eye alt 1,176m. DigitalGlobe 2018.

We conducted a cross-sectional, population-based cohort study in an urban centre and a rural district in Uganda. The urban sample was drawn from Kampala, the capital city, which had a population of 1.5 million in an estimated 416 070 households in 2014.^11^ The rural sample was drawn from Nakaseke, a health district covering 43 167 households with an estimated population of 208 500.^11^^,^^12^ Nakaseke, which is located 50 km from downtown Kampala and 14 km from the nearest highway, has been defined as rural by the Uganda Bureau of Statistics (Fig. 1).

Twenty-five enumeration areas each were selected for the urban and rural setting, with the probability of selection proportional to the population size of the enumeration area, and 1000 adults were randomly sampled in each setting. Fieldworkers conducted home visits between November 2015 and June 2016 to assess eligibility and obtain informed consent. Inclusion criteria included: (i) age 35 years or older; (ii) full-time residency in either Kampala or Nakaseke; and (iii) capacity to consent to the study. Exclusion criteria included having active pulmonary tuberculosis or a current respiratory infection and pregnancy. The study was approved by the institutional review boards of Mulago Hospital and the Uganda National Council of Science and Technology in Kampala, Uganda and the Johns Hopkins School of Medicine in Baltimore, United States of America.

Anthropometric measurements were taken in triplicate. Spirometry was carried out using an Easy on-PC spirometer (ndd, Zürich, Switzerland) and participants with low-quality results were asked to repeat the test on another day for up to three attempts.^14^ Those with evidence of pulmonary obstruction were tested again after administration of a bronchodilator (i.e. 400 µg of inhaled salbutamol). As there were no established reference parameters for lung function among Ugandans, we used the reference for African American individuals in the United States’ National Health and Nutrition Examination Survey.^15^ We defined pulmonary obstruction as a pre-bronchodilator ratio of forced expiratory volume in 1 second (FEV_1_) to forced vital capacity (FVC) below the lower limit of normal (i.e. a *z*-score ≤ −1.64).

We investigated four chronic respiratory conditions: (i) COPD, defined as a post-bronchodilator FEV_1_/FVC ratio *z*-score ≤ −1.64;^16^ (ii) a restrictive spirometry pattern, defined as a pre-bronchodilator FVC *z*-score ≤ −1.64 with a post-bronchodilator FEV_1_/FVC ratio *z*-score > −1.64;^17^ (iii) asthma, defined as a self-reported wheeze, use of asthma medication in the previous 12 months or a physician’s diagnosis of asthma;^18^ and (iv) chronic bronchitis, defined as self-reported phlegm production for at least 3 months each year in two successive years.^19^ In addition, we examined airflow-limitation reversibility, defined as a post-bronchodilator increase in FEV_1_ of 12% or more from baseline or an increase in FVC of 200 mL or more.^16^ Respiratory symptoms and functional status were evaluated using the St. George’s Respiratory Questionnaire, version 1 of the 36-item short-form health survey (SF-36) and a modified BOLD (Burden of Obstructive Lung Disease) questionnaire,^10^^,^^20^^,^^21^ all adapted to the local language (i.e. Luganda).

Demographic questionnaires asked for details of biomass fuel smoke exposure, human immunodeficiency virus (HIV) infection and history of treatment for pulmonary tuberculosis. Individuals in households that used wood or charcoal for cooking or heating were regarded as being exposed to biomass fuel smoke. Daily smoking was defined as self-reported smoking of one or more cigarettes per day, underweight, as a body mass index (BMI) < 18.5 kg/m^2^ and obesity, as a BMI ≥ 30 kg/m^2^.

### Biostatistical methods

Our primary aims were to determine how the age- and sex-adjusted prevalence of chronic respiratory diseases varied according to place of residence (i.e. urban or rural) and to estimate the fraction of chronic respiratory disease that could be attributed to urbanization. Risk factors for chronic respiratory conditions were identified using single variable and multivariable logistic regression models; as risk factors, we included sex, age, place of residence, daily smoking, use of biomass fuels, history of treatment for pulmonary tuberculosis, history of HIV infection and BMI. The adjusted odds ratios from these models were used to calculate attributable fractions for different risk factors in our study population and represent the proportional reduction in morbidity that would result if exposure to a particular risk factor were removed.^22^ Associations between these risk factors, FEV_1_, FVC and FEV_1_/FVC ratio *z*-scores were evaluated using multivariable linear regression models. In addition, multivariable logistic regression was used to analyse the association between reversibility and these risk factors. For other analyses, a *χ^2^* or Fisher’s exact test was used to compare proportions between groups and a *t* test, analysis of variance or Wilcoxon rank–sum test was used to compare continuous variables between subgroups, as appropriate. All analyses were carried out in R (The R Foundation, Vienna, Austria) and we used the epitools package to perform direct standardization by age using population structure data for 2015 from the Uganda Bureau of Statistics.^11^^,^^23^^,^^24^

## Results

Of the 2000 individuals originally identified for enrolment in the study, 1772 met inclusion criteria, consented and agreed to undergo spirometry. Ultimately, 84.8% (1502/1772) had acceptable and reproducible spirometry results (Table 1): their average age was 46.9 years (standard deviation, SD: 10.6) and 46.5% (689/1502) were male. Self-reported biomass fuel smoke exposure was high in both rural and urban settings; in rural settings, 99.6% (832/837) of individuals reported using biomass fuels for cooking. The type of biomass fuel varied: 92.3% (771/837) of rural participants reported using wood, whereas 86.0% (564/665) of urban participants reported using charcoal. We did not find any difference between the settings in self-reported history of HIV infection or treatment for pulmonary tuberculosis or in household size. However, a significantly greater percentage of participants in the urban setting had a secondary education (*P* < 0.001).

**Table 1:** Study participants, chronic respiratory disease in rural and urban Uganda, 2015–2016

Characteristic	Rural sample (*n* = 837)	Urban sample (*n* = 665)	*P*
**Age in years, mean (SD)**	49.1 (11.2)	44.1 (8.9)	< 0.001
**Male sex, no. (%)**	380 (45.4)	318 (47.8)	0.40
**Height in m, mean (SD)**	1.60 (0.1)	1.62 (0.1)	< 0.001
**BMI, mean (SD)**	24.0 (4.5)	25.9 (5.4)	< 0.001
**Secondary education, no. (%)**	173 (21)	328 (50)	< 0.001
**Household size, median (IQR)**	5 (3–7)	5 (3–7)	0.50
**Biomass fuel smoke exposure, no. (%)**			
Daily biomass fuel use	832 (99.6)	614 (93.6)	< 0.001
Fuel type			
Wood	771 (92.3)	50 (7.6)	< 0.001
Charcoal	61 (7.3)	564 (86.0)	< 0.001
Kerosene	1 (0.1)	22 (3.4)	< 0.001
Propane	0 (0.0)	12 (1.8)	< 0.001
Electricity	2 (0.2)	6 (0.9)	0.16
Other	0 (0.0)	2 (0.3)	0.38
**Daily tobacco smoking, no. (%)**	66 (7.9)	65 (9.8)	0.20
**Clinical history, no. (%)**			
Personal history of treatment for pulmonary tuberculosis	22 (3.0)	22 (3.6)	0.70
Personal history of hypertension	80 (9.8)	77 (13.3)	0.05
Personal history of diabetes	5 (0.7)	22 (4.5)	< 0.001
Personal history of HIV infection	68 (8.1)	65 (9.8)	0.67
Family history of asthma or COPD	6 (1.0)	137 (33.6)	< 0.001

BMI: body mass index; COPD: chronic obstructive pulmonary disease; HIV: human immunodeficiency virus; IQR: interquartile range; SD: standard deviation.

### Prevalence and risk factors

**Fig. 2:**
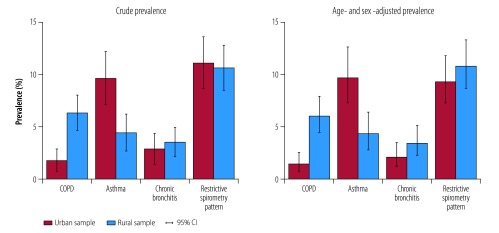
Crude and age- and sex-adjusted prevalence of chronic respiratory disease in rural and urban Uganda, 2015–2016

CI: confidence interval; COPD: chronic obstructive pulmonary disease.

Note: Age- and sex-adjusted values were calculated using mid-year estimates for 2015 from the Uganda Bureau of Statistics.

The overall age- and sex-adjusted prevalence of the four chronic respiratory conditions studied was 20.2%: 20.8% in the rural and 19.4% in the urban setting (*P* = 0.54). For COPD, the adjusted prevalence was 6.1% in rural participants versus 1.5% in urban participants (*P* < 0.001). In contrast, asthma was more prevalent in urban participants, in whom the adjusted prevalence was 9.7% compared with 4.4% in rural participants (*P* < 0.001; Fig. 2). The adjusted prevalence of chronic bronchitis was similar in rural and urban participants (3.5% versus 2.2%, respectively; *P* = 0.62), as was that of a restrictive spirometry pattern (10.9% versus 9.4%, respectively; *P* = 0.82).

**Fig. 3:**
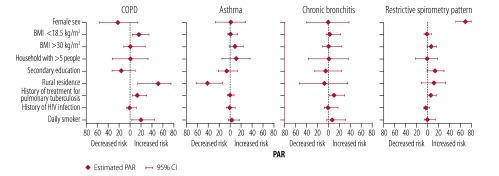
Risk factors for chronic respiratory conditions, Uganda, 2015–2016

BMI: body mass index; CI: confidence interval; COPD: chronic obstructive pulmonary disease; HIV: human immunodeficiency virus; PAR: population attributable risk.

Note: The four chronic respiratory conditions considered were chronic obstructive pulmonary disease (COPD), a restrictive spirometry pattern, asthma and chronic bronchitis.

The most important factor associated with COPD was living in a rural environment, followed by daily smoking, a BMI < 18.5 kg/m^2^ and a history of treatment for pulmonary tuberculosis. Specifically, 51.5% (95% confidence interval, CI: 13.5–75.8) of the prevalence of COPD was attributable to living in a rural environment, 19.5% (95% CI: 3.1–45.2) was attributable to daily smoking, 16.0% (95% CI: 4.3–35.1) was attributable to a BMI < 18.5 kg/m^2^ and 13.0% (95% CI: 3.5–30.8) was attributable to a history of treatment for pulmonary tuberculosis (Fig. 3). For asthma, urban residence was the principal risk factor, with a PAR of 41.4% (95% CI: 14.2–63.1). The PAR of a history of treatment for pulmonary tuberculosis was 9.5% (95% CI: 0.8–29.8) for chronic bronchitis and 5.4% (95% CI: 0.2–16.4) for a restrictive spirometry pattern.

### Symptoms and functional status

Although the reported rate of wheezing in the past 12 months was similar in urban and rural participants, the rate of asthma-related medication use and of a previous diagnosis of asthma were significantly higher in urban participants (Table 2). In addition, the reported number of days of work missed due to respiratory disease and the proportion of participants shortness of breath were both higher in the urban setting. There was no significant difference between the groups in respiratory symptoms, as assessed using the St. George’s Respiratory Questionnaire (Table 3). Participants from the urban setting reported fewer impairments in emotional well-being than those from the rural setting: the mean SF-36 score was 57.2 and 77.3 points, respectively (*P* = 0.001). There was no difference in physical functioning, social functioning, role limitation because of emotional problems, or overall health between the groups.

**Table 2:** Chronic respiratory disease symptoms, by rural or urban residence, Uganda, 2015–2016

Symptom	Rural sample (*n* = 837)	Urban sample (*n* = 665)	*P*
	No. (%)	No. (%)	
**Asthma**			
Self-reported wheezing or whistling in the chest in the past 12 months	47 (5.6)	39 (5.9)	0.99
Self-reported medication use to aid breathing in the past 12 months	17 (2.0)	51 (7.7)	< 0.001
Previous diagnosis of asthma	7 (0.8)	16 (2.4)	0.04
**Chronic bronchitis**			
Self-reported coughing up sputum for at least 3 months in each of two successive years	27 (3.2)	16 (2.4)	0.41
**Functional status**			
Self-reported experience of missing work due to, or having daily activities impeded by, respiratory problems in the past 12 months	7 (0.8)	26 (3.9)	< 0.001
Self-reported shortness of breath on physical exertion	73 (8.7)	154 (23.2)	< 0.001

**Table 3:** Disease severity and health-related quality of life, study participants with chronic obstructive pulmonary disease in rural and urban Uganda, 2015–2016

Parameter	Rural participants with COPD^a^ (*n* = 53)	Urban participants with COPD^a^ (*n* = 12)	*P*
**COPD severity,^b^ no. (%)**			0.04
Mild	20 (37.7)	3 (25.0)	ND
Moderate	23 (43.4)	8 (66.7)	ND
Severe	10 (18.9)	0 (0.0)	ND
Very severe	0 (0.0)	1 (8.3)	ND
**St. George’s Respiratory Questionnaire score,**^20^ **mean (95% CI)**			
Symptoms domain	48.3 (40.5–56.1)	63.2 (51.6–74.8)	0.07
Activity domain	37.4 (26.8–48.0)	34.1 (17.8–50.5)	0.76
Psychosocial impact domain	27.4 (19.0–35.9)	32.0 (21.4–42.7)	0.59
Total	34.6 (25.5–43.6)	38.1 (27.4–48.7)	0.70
**MRC dyspnoea scale score, median (IQR)**	2 (1–2)	2 (2–2)	0.12

CI: confidence interval; COPD: chronic obstructive pulmonary disease; IQR: interquartile range; MRC: Medical Research Council; ND: not determined.

^a^ Chronic obstructive pulmonary disease (COPD) was defined as a post-bronchodilator ratio of forced expiratory volume in 1 second (FEV_1_) to forced vital capacity (FVC) below the lower limit of normal (i.e. *z*-score ≤ 1.64) of the African American reference population in the United States’ National Health and Nutrition Examination Survey (NHANES).

^b^ COPD severity was defined by the GOLD criteria as mild (i.e. FEV_1_ ≥ 80% predicted), moderate (i.e. 50% ≤ FEV_1_ < 80% predicted), severe (i.e. 30% ≤ FEV_1_ < 50% predicted) and very severe (FEV_1_ < 30% predicted).^25^

### Lung function

**Fig. 4:**
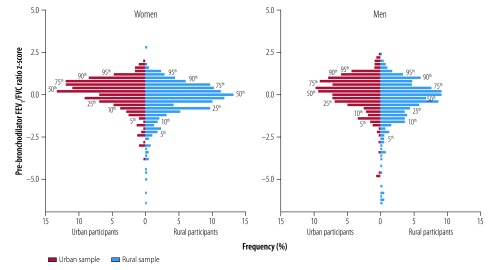
Lung function, by sex and place of residence, Uganda, 2015–2016

**Fig. 5:**
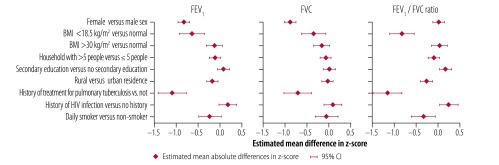
Estimated effect of sociodemographic and clinical variables on lung function, Uganda, 2015–2016

BMI: body mass index; CI: confidence interval; FEV_1_: forced expiratory volume in 1 second; FVC: forced vital capacity; HIV: human immunodeficiency virus.

Notes: Mean absolute differences in *z*-scores and corresponding 95% CI were obtained from multivariable regression analysis. A normal BMI was in the range 20–25 kg/m^2^.

In all participants, the mean ± SD pre-bronchodilator FEV_1_, FVC and FEV_1_/FVC ratio was 2.53 ± 0.66 L, 3.18 ± 0.75 L and 0.79 ± 0.08, respectively. Fig. 4 presents the distribution of the pre-bronchodilator FEV_1_/FVC ratio *z*-score, stratified by place of residence and sex. Both female and male participants in the rural setting had a lower FEV_1_/FVC ratio than their urban counterparts. Among participants found to have pulmonary obstruction on pre-bronchodilator spirometry, the proportion with a post-bronchodilator FEV_1_ less than 50% of predicted was higher in the rural than the urban setting: 18.9% (10/53) versus 8.3% (1/12), respectively. However, the difference was not significant (*P* = 0.67). Fig. 5 shows the estimated effect of sociodemographic and clinical variables on the pre-bronchodilator FEV_1_, FVC and FEV_1_/FVC ratio, as determined by multivariable regression analysis. A history of treatment for pulmonary tuberculosis was associated with a low adjusted pre-bronchodilator FEV_1_ (p<0.001) FVC (*P *< 0.001) and FEV_1_/FVC ratio (*P* < 0.001); living in a rural environment was associated with a low adjusted pre-bronchodilator FEV_1_ (*P* = 0.009) and FEV_1_/FVC ratio (*P* < 0.001); and self-reported daily tobacco smoking was associated with a low adjusted pre-bronchodilator FEV_1_/FVC ratio (*P* = 0.001). Among participants with obstruction, 26.2% (17/65) exhibited reversibility on post-bronchodilator spirometry – the proportion was 50.0% (6/12) in the urban setting and 20.8% (11/53) in the rural setting (*P* = 0.065).

FEV_1_: forced expiratory volume in 1 second; FVC: forced vital capacity.

Notes: The figure shows pre-bronchodilator FEV_1_/FVC ratio *z*-score distribution. Each bar represents a 0.2 increment in *z*-score. Bars that include the 5^th^, 10^th^, 25^th^, 50^th^, 75^th^, 90^th^ or 95^th^ percentile in each distribution are labelled accordingly. The *z*-scores were calculated using data for the African American reference population in the United States’ National Health and Nutrition Examination Survey (NHANES).

## Discussion

Although previous studies in Uganda and sub-Saharan Africa have examined the prevalence of, and risk factors for, single respiratory diseases,^8^^–^^10^ few have examined the effect of urbanization on a range of chronic respiratory diseases and none has assessed the PAR. We found that one in every five adults in our study had a chronic respiratory condition and that place of residence was the single most important PAR factor. Specifically, 40% of people with asthma were attributable to urban residence and 50% of people with COPD were attributable to rural residence (a proxy for daily wood smoke exposure), which was a more important factor than tobacco smoking. Many participants in both urban and rural settings had a low lung volumes – this was associated with a history of treatment for pulmonary tuberculosis and being underweight, both of which are common in low- and middle-income countries.

Published data on the prevalence of chronic respiratory diseases in low- and middle-income countries cover a wide range. In the BOLD study, the prevalence of COPD in primarily urban environments ranged from 11.4 to 23.8% and that of a restrictive spirometry pattern ranged from 4.2 to 48.7%.^10^^,^^26^ In Uganda, a FRESH AIR study found a COPD prevalence of 16.2% in a rural setting.^8^ The prevalence in our study was lower: 6.1% in rural and 1.5% in urban participants. There are several possible reasons for the varying estimates: (i) there may have been differences between the studies in the spirometry criteria used to diagnose COPD;^27^ (ii) the proportion of tobacco smokers was higher in the FRESH AIR study, which covered a tobacco-growing region, than in our study (36% versus 9%, respectively) or in other parts of Uganda;^8^^,^^28^ and (iii) the average age of participants was lower in our study than in the BOLD study: 47 versus 56 years, respectively.^10^ The difference in estimates is unlikely to have been due to differences in the proportion of participants with acceptable spirometry between our study (84.8%) and either BOLD (93%) or FRESH AIR (97%).^8^^,^^10^

The prevalence of chronic bronchitis in our study was 3.3%, which was similar to that in the BOLD study (i.e. 3.1%) and in a population-based study in South Africa (i.e. 2.3 to 2.8%).^29^^,^^30^ We found no difference between urban and rural settings, possibly because both ambient air pollution exposure in urban Kampala and household air pollution exposure in rural Nakaseke were high.^31^ The prevalence of wheezing and of a physician’s diagnosis of asthma were similar in our study and in a population-based study in sub-Saharan Africa.^32^ Further, the overall prevalence of asthma was higher in our urban sample than our rural sample, which is consistent with findings in both high- and low-income regions, including sub-Saharan Africa.^33^^,^^34^

Urban populations, particularly in low- and middle-income countries, are exposed to several risk factors that predispose to smaller lungs and accelerated lung function decline.^35^ Early life events, such as childhood respiratory infection,^36^ micronutrient deficiency and both ambient and household air pollution,^37^ can influence lung size and function, thereby predisposing individuals to obstructive pulmonary disease in adulthood.^38^ High cortisol and inflammatory biomarker levels due to urban stressors have been linked to lung injury.^39^ Exposure to indoor and outdoor allergens, pollutants and irritants during the early years of life may tip a child’s immune system’s response from a non-asthma to an asthma phenotype, thereby leading to adult asthma. Diminished early exposure to parasites (exposure results in parasite-induced immunoglobulin-E) may lead to the development of atopic disease.^40^ Greater exposure to additives and synthetic foods may influence nutrition, which has been associated with asthma.^35^ A higher risk of communicable diseases such as tuberculosis and recurrent pulmonary infection can predispose to chronic respiratory diseases such as COPD, restrictive lung disease, asthma, chronic bronchitis and bronchiectasis.^30^^,^^34^^,^^41^ Our findings reflect the effect of exposure to these risk factors on airway and parenchymal injury and disease and are consistent with previous data on the distribution of chronic respiratory disease in urban and rural environments in low- and middle-income countries.^33^^,^^34^^,^^41^

The leading risk factor for several chronic respiratory diseases in both urban and rural settings remains chronic inflammation due to exposure to environmental substances. Although cigarette exposure is low in many African countries, it is expected to rise with growing urbanization over the next two decades, thereby increasing the prevalence of chronic respiratory disease.^2^ Today, household air pollution remains the leading cause of COPD.^28^^,^^42^ In our study, biomass fuel was commonly used for cooking at both rural and urban sites though the type of fuel was different: the primary fuel was wood at rural sites and charcoal at urban sites. We were unable to assess differences in the relative risk of different types of fuel. However, burning wood emits more particulate matter than burning charcoal or liquefied petroleum gas and there have been reports that respiratory symptoms are more common in individuals in low- and middle-income countries who use wood than in those who use charcoal or liquefied petroleum gas.^38^

Our study has several strengths. First, it had a large population-based design and comprehensive spirometry and respiratory data were collected. Second, we used the lower limit of normal for African American individuals in the National Health and Nutrition Examination Survey to identify COPD and a restrictive spirometry pattern, this helped prevent overdiagnosis of obstruction and restriction, particularly in elderly people.^27^ Third, by calculating PARs, we were able to gain a better understanding of the risk factors for chronic respiratory diseases. Our study also has some potential shortcomings. First, we were unable to recruit 15.2% (270/1772) of individuals who met inclusion criteria after two follow-up visits; nevertheless, our non-response rate was lower than in many other studies. Second, participants who had incomplete spirometry results were predominantly elderly or had severe respiratory disease, which highlights the difficulty of spirometry in those with reduced pulmonary function. The disease burden may therefore, have been underestimated. Third, urban participants were younger than rural participants, which may have affected the crude disease prevalence; however, we calculated age- and sex-adjusted prevalences to reduce the risk of bias. Fourth, we defined asthma as a self-reported wheeze, use of asthma medications or a physician’s diagnosis. The last two criteria are closely linked to health-care access and utilization, which may be influenced by socioeconomic status. Consequently, the higher rates we observed for these two criteria among urban participants may reflect better access to health care. However, post-bronchodilator reversibility, which indicates greater airway reactivity, was significantly more common in urban residents with obstruction. Fifth, we did not assess individual exposure to air pollution. Better understanding of the association between urbanization and chronic respiratory disease requires longitudinal assessment of individual exposure to air pollution.

The increase in urbanization over the next two decades is expected to occur almost exclusively in low- and middle-income countries and, by 2030, half of sub-Saharan Africa’s population will reside in an urban area.^7^ However, poor infrastructure and a lack of policy interventions aimed at sustainable urban growth will have severe health implications for many people in low- and middle-income countries worldwide. Future public health interventions will depend on a good understanding of the evolving disease burden. Currently, chronic respiratory disease is associated with chronic inflammation throughout life, whether due to household air pollution, the urban microenvironment (e.g. allergens and outdoor air pollution) or recurrent pulmonary infection.^40^ As urban dwellers transition to cleaner fuels for cooking and heating, cigarette exposure and outdoor air pollution will become increasingly important risk factors.

In summary, we found a high burden of chronic respiratory disease in both rural and urban Uganda. Place of residence was the single most important risk factor: urban residence was the principal risk factor for asthma and rural residence was the principal risk factor for COPD. As urbanization continues in sub-Saharan Africa, the profile and burden of chronic respiratory disease are likely to change in parallel.
